# Guest editor’s editorial: BDQ Special Issue — “Liquid Biopsy & Next Generation Biomarkers”: Published in conjunction with the 9th Gene Quantification Event in Freising Weihenstephan, Germany www.qPCR-dPCR-NGS-2019.net

**DOI:** 10.1016/j.bdq.2019.100086

**Published:** 2019-03-22

**Authors:** Michael W. Pfaffl

**Affiliations:** Animal Physiology & Immunology, School of Life Sciences, Technical University of Munich, Weihenstephaner Berg 3, 85354, Freising, Germany

Dear reader,

I had the honour to edit another special issue in our young scientific journal ‘Biomolecular Detection and Quantification’. It is entitled ‘*Liquid Biopsy & Next Generation Biomarkers*’, published in conjunction with 9^th^ Gene Quantification Event (www.qPCR-dPCR-NGS-2019.net), and therefore covering similar key topics. The publications were also presented as oral presentations at the scientific symposium, which took place from 18. to 22. March 2019 in Freising Weihenstephan at the School of Life Sciences Weihenstephan, Technical University of Munich.

The methodological focus of the conference and the topics herein are all based on nucleic acids quantification methods and their applications in molecular research or clinical diagnostics. Special focus was made on liquid biopsies, micro-genomics, single-cells applications, or new matrices like extracellular vesicles for the development of advanced next generation biomarkers. Hence the biomarker development should aim in reproducible and valid biomarker signatures, which are capable of revealing specific biological traits in the probands or should highlight molecular changes, according to a disease status or pathological conditions [1]. The next generation biomarkers could be the essence of the integrative analysis of various ‘-omic’ levels considering their molecular interactions, e.g. between mRNA – microRNA – lncRNA or transcripts - proteins - function. Final goals are deeper insights into the molecular and cellular interaction of disease mechanisms or physiological- and pathological pathway dynamics.

Therefore all sensitive and highly sophisticated molecular quantitative techniques, like quantitative real-time PCR (qPCR), digital PCR (dPCR) and all varieties of next generation sequencing (NGS) methods are in the scientific focus. Also the optimization and standardization of mentioned methods comes hand-in-hand with reproducible and reliable quantitative results [2]. Today the multitude of generated data, especially from the holistic and high-throughput technologies, are often unmanageable and incomprehensible for the researcher. These datasets need to be filtered, sorted and connected with relevant biological pathways and physiological questions. Therefore the generation of big-data must come together with complex data-analysis and newest bioinformatical software applications [1]. The application of multi-omics, the combination of high-throughput methods with intelligent data integration and the usage of meaningful bioinformatical tools seems to be an essential key step on the way to discover the next generation of biomarker signatures [3].

A further challenge in clinical molecular diagnostics is the real effective time from sampling-to-result, which might be essential for future applications directly at the bedside or far out in the field. Hence fast diagnostic methods in small mobile devices need to be developed. On the other hand also the necessity of reproducibility and validity is very important for a reliable biomarker signature measurement. Real-time PCR with its almost unlimited potential of nucleic-acid amplification in combination with high speed seems to be the future method of choice. Single marker RNA and DNA can be rapidly amplified tube-by-tube or multiplexed to generate such biomarker signatures in less than a minute.

The clinical focus of today’s research in biomarker discovery is directed to ‘liquid biopsies’, mainly connected to circulating free DNA (cfDNA) or extracellular RNA (exRNA) based ‘circulating biomarkers’. Circulating nucleic acids are considered as stable and float in blood stream, since they are protected and bound to proteins, associated to extracellular microvesicles (EV), or fully covered by exosomal bilayer membrane. The most prominent studied EV family are exosomes (40–200 nm diameter membranous vesicles of endocytic origin), beside other EV families like microvesicles or larger membranous vesicles (50–1000 nm diameter) that are shed directly from the plasma membrane, and apoptotic blebs (200–5000 nm diameter) [4]. EV and in particular exosomes contain a multitude of protected microRNA, regardless which all three are foreseen to have high potential in today’s molecular diagnostics. They intend to be applied in clinical testing for early diagnosis, to distinguish between sick individuals from healthy probands, or in disease stratification and classification of cancer. Liquid biopsies are assumed to be non- or at least minimal invasive and the easy sampling of these specific circulating biomarkers has encouraged intensive cfDNA and microRNA biomarker research. So far circulating extracellular vesicles protecting microRNAs and exRNA have been detected in the majority of body fluids, e.g. blood, urine, milk, sweat, saliva, tears, ejaculate, or cerebrospinal fluid [5].

Herein we present the potentials of nucleic acid diagnostic in liquid biopsy. Blood derived cfDNA can serve as a surrogate marker for multiple indications in cancer patients, including the diagnosis, prognosis and the decease monitoring (Bronkhorst et al.). The review highlights the importance of the pre-analytical diagnostic steps and refine the current cfDNA analysis strategies. It further discusses how the general understanding in cfDNA can be improved with focus on its origin, physical properties, and circulation dynamics.

In a further publication by Johansson et al. important considerations for the cfDNA detection in human plasma are discussed. Focus is on the optimization of the molecular diagnostics workflow of cfDNA quantification, and how each experimental step can be easily validated by using qPCR, including the problematic fields of DNA contaminations, PCR inhibition, assay performance, fragment size, and target sequence. As usual and initiated by the MIQE guidelines [6,7], a step-by-step quality control through the analytical workflow is essential for a reliable and valid quantitative answer, which might be later implemented in the clinical routine.

The next focus was laid on EVs. The comparison and validation of various EVs isolation methods from human urine, an upcoming matrix for non-invasive liquid biopsies, is reported by Mussack et al. This study describes urinary microRNA and was performed in strict adherence with the MISEV guidelines (minimal information for studies of extracellular vesicles) [8]. Compliancy was demonstrated by a broad evaluation spectrum of biophysical and proteomic EV characteristics alongside with transcriptomic results.

In a second extracellular RNA related study, the normalization properties of urinary derived stable mRNA are introduced and discussed. They could provide useful information about cellular transcription rate in urogenital tissues, which could possibly be used in the future as biomarkers or normalizers in the urine supernatant (Gunasekaran et al.).

Millington et al. are presenting a rapid qPCR methodology in the context of molecular diagnostics. An ultra-fast qPCR setup for short DNA fragments is reported capable of amplifying DNA in less than 15 s. This seems to be the future of nucleic-acid based clinical diagnostics for DNA detection and quantification.

Circular RNAs (circRNAs) are a new family in the wide class of non-coding RNAs. Due to their circular structure they are assumed to be more stable, compared to their linear RNA counterparts, and may serve in future as stable diagnostic biomarkers (Preusser et al.). Herein the need of essential quality controls and criteria for the characterization and validation of circRNA are discussed in the context of high-throughput sequencing.

Last but not least, applied research is presented with the development of event-specific qPCR and ddPCR detection method for the genetically modified alfalfa (Gürtler et al.). To test for robustness of the presented quantitative assays, various real-time PCR instruments were implemented in the development phase. PCR conditions, assay sensitivity and specify were shown down to 30 DNA copies per setup. GMO assay validation results were reported to be in line with the “Minimum Performance Requirements for Analytical Methods of GMO Testing” of the European Network of GMO Laboratories [9].

I hope the topic selection of the presented publications in this first special issue has attracted your attention and will help you to solve the analytical challenges in your own biomarker discovery study. A second part of this ‘Liquid Biopsy & Next Generation Biomarkers’ issue will be published later this year. In this addendum the focus will laid on ‘digital PCR’, on how to multiplex in dPCR, and on biometrology. What is biometrology and what could it mean for biomolecular research?

Further topics are new data analysis methods, e.g. what is the advantage of an isomiR analysis of next generation small-RNA sequencing big data, and how to apply artificial intelligence (AI) for qPCR data analysis. As guest editor I am looking forward for this innovative new research topics.

To support the present published written articles, we provide free access to around 400 recorded talks from the past years via our streaming portal eConferences (eConference.qPCR-dPCR-NGS-2019.net). The streaming portal is dedicated to all scientists with interest in qPCR, dPCR, NGS, MicroGenomics, and Molecular Diagnostics. You can stream all recorded talks presented at the latest symposium 9^th^ Gene Quantification Event, taking place at TUM Weihenstephan in March 2019, and older events going back to the qPCR Symposium 2010 in Vienna. We provide the presentations for free via movie streaming technology in high quality, high resolution and perfect sound quality in high speed.

Enjoy reading or the special issue and watching our eConference.
